# The natural iron chelators' ferulic acid and caffeic acid rescue mice's brains from side effects of iron overload

**DOI:** 10.3389/fneur.2022.951725

**Published:** 2022-10-14

**Authors:** Mahdi AAlikhani, Masoumeh Khalili, Mehrdad Jahanshahi

**Affiliations:** ^1^Department of Medical Biotechnology, School of Advanced Medical Technologies, Golestan University of Medical Sciences, Gorgan, Iran; ^2^Neuroscience Research Center, Golestan University of Medical Sciences, Gorgan, Iran; ^3^Infectious Diseases Research Center, Golestan University of Medical Sciences, Gorgan, Iran; ^4^Department of Anatomy, Neuroscience Research Center, Faculty of Medicine, Golestan University of Medical Sciences, Gorgan, Iran

**Keywords:** iron overload, neuro-cerebral diseases, neurogenic disorders, natural products, ferulic acid, caffeic acid, antioxidant enzyme activity

## Abstract

Studies have shown that iron accumulation in the brain leads to neurogenic disorders. Novel iron chelating agents such as natural remedies are useful to decrease the side effects of iron in the brain. In addition, flavones and polyphenols are capable of chelating metals. In the current study, we evaluated the iron chelating capacity of ferulic acid and caffeic acid in the brain tissues of iron-overloaded mice. The mice received iron dextran intraperitoneally four times a week for 6 weeks. Next, blood samples were taken from the mice. In addition, brain tissues were excised for tissue staining as well as total iron and catalase (CAT) activity assessment. Ferulic acid and caffeic acid significantly decreased iron content in both brain and serum samples. Ferulic acid decreased iron by 50 and 51% more than the iron dextran-treated mice and by 43 and 2% more than desferal (DFO)-treated mice in serum and brain, respectively. In addition, caffeic acid reduced iron 57% more than the iron-treated group and 49 and 2% more than the desferal-treated group in the serum and brain, respectively. The catalase activity decreased with the increase in iron. By administering natural compounds, the catalase activity was increased equal to that of the control group. Thus, ferulic acid and caffeic acid might be possible natural iron chelators for brain iron overload therapy.

## Introduction

Iron is an essential element for the survival of organisms (1, 2). The brain needs iron for many important functions, such as myelination, neurotransmitter synthesis, nitric oxide metabolism, and other biochemical activities (3). The brain is very sensitive to oxidative stress caused by iron overload (4). Various studies have demonstrated that iron overload leads to brain iron overload and ultimately affects brain function. Iron overload in intracellular parts or certain areas of the brain leads to various neuro-cerebral diseases (5). Heavy metal imbalances such as Fe, Cu, and Zn lead to oxidative stress, which is the key to many brain diseases (4, 6). For instance, increased levels of these metals are observed in specific areas of the brain in Alzheimer's and Parkinson's disease, and other brain diseases (2, 7, 8). Brain iron homeostasis is very important and its hippocampal deposition affects memory and learning (3). Brain iron overload triggers the Fenton reaction and the production of free radicals. As a result, free radicals damage membrane lipids, proteins, and nucleic acids, thereby affecting neuronal function and increasing apoptotic marker levels (9).

Many diseases, as well as premature aging of the body, are caused by the increased concentration of free radicals, which cause oxidation and destruction of normal cells. Studies have shown that iron chelators protect neurons from the negative effects of iron in a mouse model of Parkinson's disease (10). The iron-chelation activity of desferal (DFO), deferiprone, and deferasirox was shown in the brain of iron-overloaded rats. They reduced iron deposition and decreased its subsequent negative effects (11, 12). Commonly, DFO and deferasirox or deferiprone are used to treat diseases related to iron accumulation. However, they have various side effects such as irritation, systemic allergic reactions such as rash, urticaria, anaphylactic reaction, angioedema, agranulocytosis, arthropathy, gastrointestinal symptoms, an increase in transaminases levels, and weight gain (13, 14). This highlights the need for further research on new and safe chelators.

Therefore, it is important to study antioxidant therapeutic strategies to improve the antioxidant capacity and decrease iron content. In addition, new therapies should not be toxic to patients with iron-overloaded conditions. Considering the side effects of common iron chelators, researchers are now interested in natural compounds. Various studies have shown that some natural compounds are iron-chelating agents *in vitro* and have the capacity to reduce the toxic effects of iron in the brain (8). Studies have shown that the extracts of some mushrooms and plants have the iron-chelation capacity *in vivo* (15, 16). Nowadays, attention has been paid to the use of natural compounds with iron-chelation capacity. Ferulic acid can be abundantly found in cereals, fruits such as apples, oranges, and bananas, and vegetables such as potatoes, tomatoes, and cabbage (17). Studies have shown that ferulic acid has antioxidant properties and protects DNA and lipids against oxidative substances with anti-diabetic, anti-inflammatory, anti-aging, and anti-cancer properties (18). Balasubashini et al. (19) have shown that pure ferulic acid inhibits lipid peroxidation in diabetic rats. Pure ferulic acid had nephroprotective and antioxidant effects on rats (7). In other studies, pure ferulic acid and caffeic acid have been shown to reduce cholesterol metabolism and had antioxidant activity in rats (20). Caffeic acid can be found in a variety of mushrooms, tea, and coffee. Researchers have reported different effects of caffeic acid, such as antiviral, antioxidant, anti-cancer, and anti-inflammatory properties (21, 22). In addition, various studies show that pure caffeic acid has antioxidant activity against ischemia-reperfusion injury, hepatoprotective ability against OXT-induced toxicity, anti-lipid peroxidation activity, and cardio-protective activity (23–25). Thus, the present study aimed to investigate the iron-chelating and antioxidant activity of two phenolic compounds, ferulic acid and caffeic acid, in the different areas of the brain (cerebellum, hippocampus, and cortex) as well as serum.

## Materials and methods

### Measurement of iron chelating capacity in *in vitro*

To measure the chelation capacity *in vitro*, 800 μg/ml of caffeic acid (PubChem CID: 102261219) or ferulic acid (PubChem CID: 445858) was prepared. Therefore, five dilutions of the natural compounds were prepared by adding 2 ml of water to 2 ml of the compound. Then, 2.8 ml of distilled water was added to 1 ml of each dilution. Then, 50 μl of iron chloride (5 mM) and 50 μl of ferrozine (5 mM) were added to each of the test tubes containing the natural compound. After 10 min, the UV absorbance was read by a spectrophotometer at 562 nm. EDTA was used as a positive control in this experiment (26). Caffeic acid and ferulic acid were purchased from Sigma Chemical Co, Germany.

### Animal study and treatment

In total, fifty-six NMRI albino male mice (aged 6–7 weeks and weighing 20–25 g) were purchased from the Pasteur Institute (Amol, Northern Iran). They were placed in cases, as four mice per cage, and maintained at a light cycle of 12 h light and 12 h dark under controlled temperature and humidity (24 ± 25°C and 45–55%, respectively). We performed all the animal experiments in terms of the ethical guidelines approved by the Ethics Committee of Golestan University of Medical Sciences, Gorgan, Iran (Approval number: IR.GOUMS.REC.1397.179). The sample size of animals was calculated according to Charan and Kantharia (27). (Note: according to the formula, we should use four animals in every group. Because of using four mice's brains to determine total iron and four mice's brains for Perl's staining; a total of 56 mice were enrolled in this study).

To iron-overload the mice, all mice except the negative control group were injected with iron dextran intraperitoneally-*i.p*. (100 mg/kg/day) for 6 weeks and 4 times a week. Then, the mice were left alone for a month to reach balance (2). The mice were then divided into groups consisting of eight mice. A: negative control group (received normal saline), B: DFO-treated group (25 mg/kg/day DFO as *i.p*. injection), C: positive control group (iron-overloaded), D: DFO-treated iron-overload group (25 mg/kg/day), E: caffeic acid-treated iron-overloaded group (30 mg/kg/day), F: caffeic acid-treated iron-overload group (50 mg/kg/day), G: ferulic acid-treated iron-overload group (30 mg/kg/day), and H: ferulic acid-treated iron-overloaded group (50 mg/kg/day) (2, 19, 28, 29). The dosage of ferulic acid was selected according to previous studies. They stated that ferulic acid is safe in various dosages up to 100 mg/kg/day (19, 29). The pure natural compounds were used four times a week for 4 weeks intraperitoneally. DFO-treated mice received DFO (25 mg/kg/day; *i.p*.) four times a week for 4 weeks (2, 8). Groups A and B received normal saline in the 3rd month. Before killing, the mice were kept hungry overnight and then anesthetized using ketamine and xylazine. Blood samples were taken directly from the mice's ventricles. Then, blood serum was isolated and stored at −20°C. To measure total iron in the brain and liver, the brain and liver tissues were washed with normal saline and placed in PBS at −20°C. Additionally, the brain and liver samples were collected in 10% formalin for histopathological and oxidant examinations (16, 30).

### Assessment of serum ferric cation

The Pars Azmoun kit was used to measure serum iron (Pars Azmoun, Tehran, Iran) (16). Then, 100 μl of serum sample or standard (TruCal U calibrator, Pars Azmoun, Tehran, Iran) was dissolved in 1,000 μl of Solution 1 (consisting of acetate buffer 800 mM, pH 4.5, and thiourea 90 mM). After 10 min, the absorbance was measured at 600 nm using a spectrophotometer. Then, 250 μl of Solution 2 (including ascorbic acid 45 mM, Ferene 0.6 mM, and thiourea 20 mM) was added to the mixture and the absorbance was re-read after 10 min. The serum ferric cation level was calculated using the following formula:


(1) $$\begin{array}{l} Iron\left(\mu moll^{-1}\right)=\left(\frac{\Delta A\;Sample}{\Delta A\;Cal}\times 187\right)\times 0.1791 \end{array}$$

### Measurement of total iron content in the brain and liver

Rebouche et al. (31) method was used to measure total iron content in the brain and liver. A total of 100 mg of tissue samples (brain or liver) were homogenized in pure water. About 10% of trichloroacetic acid was added to 400 ml of 1 N HCl and tissue samples were homogenized. Samples were then incubated for 1 h at 95°C. After being cooled, the samples were shaken and then centrifuged at 10,000 g for 10 min. Then, 600 μl of 0.508 mM ferrozine, 1.5 mM sodium acetate, and 1% acetyl glycol was added to 200 μl of the supernatant. After 30 min of incubation at room temperature, the absorbance was read at 562 nm (31).

### Preparation of the brain and liver tissue samples

Mice were anesthetized using ketamine (90 mg/kg, *i.p*.) and xylazine (10 mg/kg, *i.p*.). The brain and liver tissues were removed and fixed in 4% paraformaldehyde. After 2 weeks, the tissues were processed using an automated tissue processing machine (Did Sabz, Urmia, Iran), and were placed in paraffin for iron-specific staining (8). A rotary microtome (Pooyan MK 1110, Iran) was used to prepare 6 μM tissue sections. The brain sections from the cerebellum, hippocampus, and cortex were used for further histopathological examinations. These sections are more related to diseases, such as Alzheimer's and Parkinson's diseases.

### Evaluation of iron deposition and histopathological examinations

The brain and liver tissues were stained using Perls' staining kit (Shimi Pajhohesh Asia, Amol, Iran). The samples were deparaffinized and dehydrated. The tissue sections were then placed in potassium ferrocyanide and ferrocyanide-hydrochloric acid solutions. Afterward, samples were washed with distilled water, juxtaposed to nuclear fast red, and then washed again. Tissues were dehydrated with 95% alcohol and absolute alcohol, respectively, and cleaned with xylazine. Finally, the tissues were covered with Entellan (Merck, Germany) and lamella glue. A × 40 light microscope (Model: BX 53, Olympus, Japan) was used to take photos from the samples with a digital camera (Model: DP73, Olympus, Japan) (8).

### Analyzing the activity of CAT

To investigate the effect of treatments on the oxidative status of the brain, liver tissues, and serum, the activity of the catalase (CAT) enzyme was measured by a ZellBio kit (ZellBio GmbH, Germany). Catalase activity was measured according to the protocol provided by the manufacturer (2).

### Statistical analysis

Data analysis was performed using a one-way analysis of variance (ANOVA) followed by the Newman–Keuls test and a *p*-value < 0.05 was considered the significant level in all tests. The data analysis was carried out using Graph Pad Prism 9.

## Results

### The iron chelation activity of ferulic acid and caffeic acid in *in vitro*

The iron-chelating capacity of ferulic acid and caffeic acid was measured *in vitro*. The results showed that the iron-chelating capacity was 61 and 58% for caffeic acid (800 μg/ml) and ferulic acid (50 μg/ml), respectively. The iron-chelating capacity of EDTA (10 μg/ml) as the standard was 93%.

### Total iron content of serum

**Figure 1:**
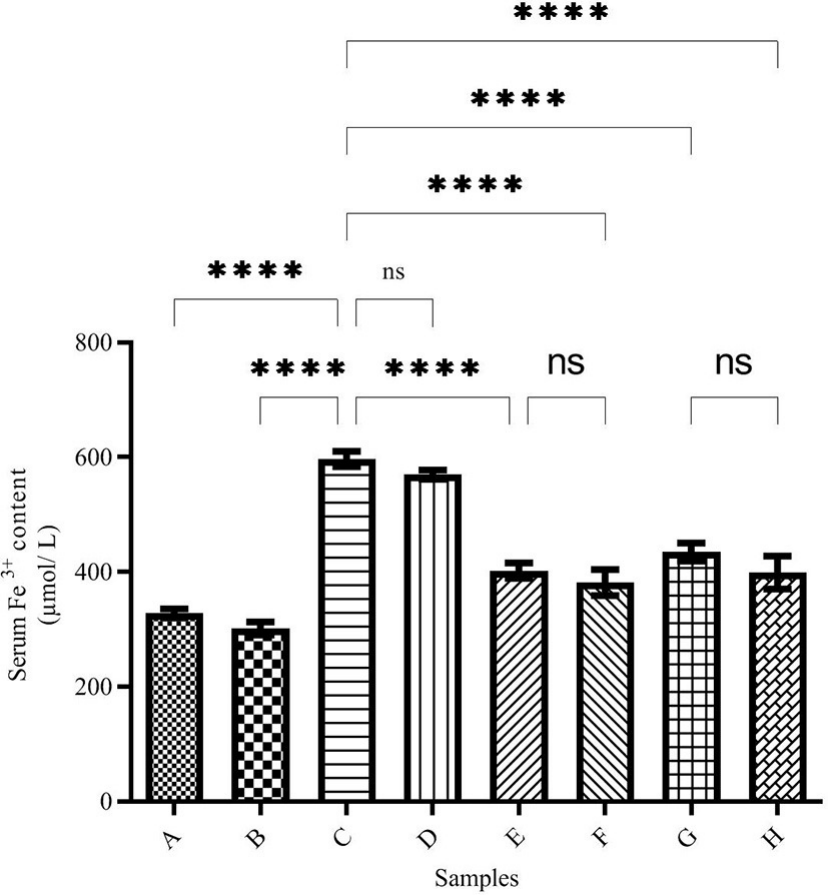
Serum Fe^3+^ content in the treated mice. Group A: control group, normal saline-receiving group; Group B: normal saline and desferal (DFO)-receiving group; Group C: iron dextran and normal saline-receiving group; Group D: iron dextran and DFO-receiving group; Group E: low dose caffeic acid-receiving group (30 mg/kg/day); Group F: high dose caffeic acid-receiving group (50 mg/kg/day); Group G: low dose ferulic acid-receiving group (30 mg/kg/day); and Group H: high dose ferulic acid-receiving group (50 mg/kg/day) (Mean ± SD; ns, non-significant and ^****^p ≤ 0.0001).

The serum iron level is shown in Figure 1. The highest serum iron level belongs to group C (the iron overloaded group). In this group, the iron level reached 597.13 ± 3.36 μmol/L, while in Group A (the negative control group), the iron level was 328.7 ± 2.63 μmol/L (*p* < 0.001). In the DFO-treated group (group B), the iron level decreased compared with group A (301.11 ± 5.45 μmol/L), but it was not significantly different from Group A. In group D (the DFO-treated iron overloaded group), the iron level was reduced significantly (569.7 ± 6.81 μmol/L), and there was no significant difference between groups C and D. In caffeic acid-treated groups (groups E and F), the iron level decreased significantly and reached 402.13 ± 6.36 and 381.6 ± 22.90 μmol/L, respectively, which was significantly lower than Group C (*p* < 0.001). In the ferulic acid-treated group, the highest iron reduction was related to Group H (398.28 ± 8.62 μmol/L).

### Total iron content of brain and liver

**Figure 2:**
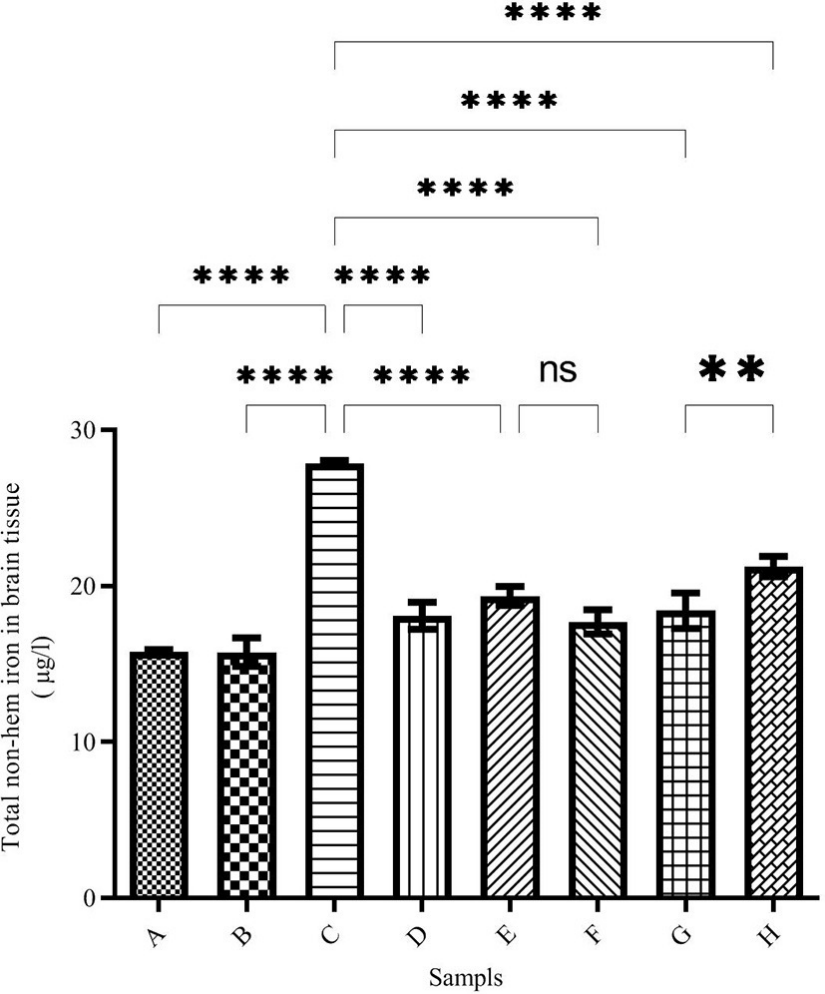
Brain total non-heme iron content in the treated mice. Group A: control group, normal saline-receiving group; Group B: normal saline and DFO-receiving group; Group C: iron dextran and normal saline-receiving group; Group D: iron dextran and DFO-receiving group; Group E: low dose caffeic acid-receiving group (30 mg/kg/day); Group F: high dose caffeic acid-receiving group (50 mg/kg/day); Group G: low dose ferulic acid-receiving group (30 mg/kg/day); and Group H: high dose ferulic acid-receiving group (50 mg/kg/day) (Mean ± SD; ^**^p ≤ 0.01 and ^****^p ≤ 0.0001).

The total brain iron was determined using the ferrozine method and the results are demonstrated in Figure 2. The highest amount of iron was seen in the brains of iron-treated mice of group C (27.85 ± 0.20 μg/L), while the total brain iron in the negative control group (group A) was 15.78 ± 0.16 μg/L. Total iron levels in the brain of DFO-treated mice were significantly reduced. The highest iron reduction was observed in the brains of caffeic acid-treated mice (50 mg/kg/day) (group F, 17.71 ± 0.76 μg/L).

**Figure 3:**
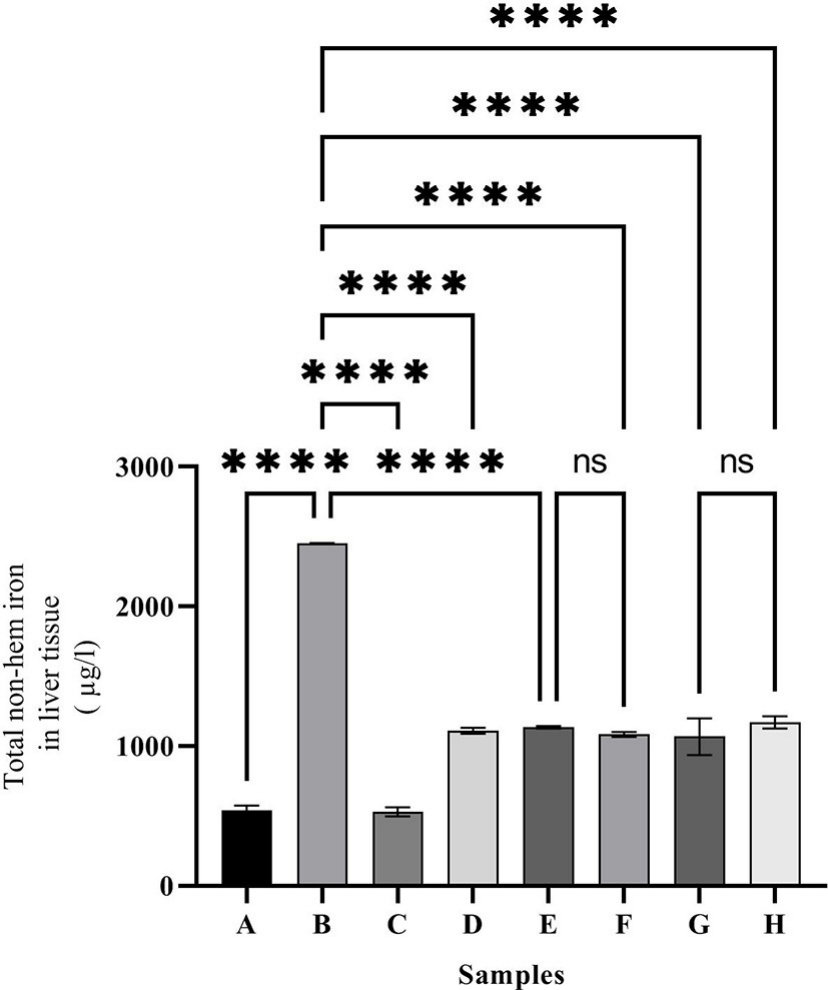
Liver non-hem iron content in the treated mice. Group A: control group, normal saline-receiving group; Group B: normal saline and DFO-receiving group; Group C: iron dextran and normal saline-receiving group; Group D: iron dextran and DFO-receiving group; Group E: low dose caffeic acid-receiving group (30 mg/kg/day); Group F: high dose caffeic acid-receiving group (50 mg/kg/day); Group G: low dose ferulic acid-receiving group (30 mg/kg/day); and Group H: high dose ferulic acid-receiving group (50 mg/kg/day) (Mean ± SD; ns, non-significant and ^****^p ≤ 0.0001).

As shown in Figure 3, the highest amount of iron in the liver was observed in group C (2,450 ± 2.26 mol/ml). Administering desferal or natural compounds significantly reduced the amount of iron in the liver tissue and brought it to the normal level equal to the control group.

### Histology parameters

**Figure 4:**
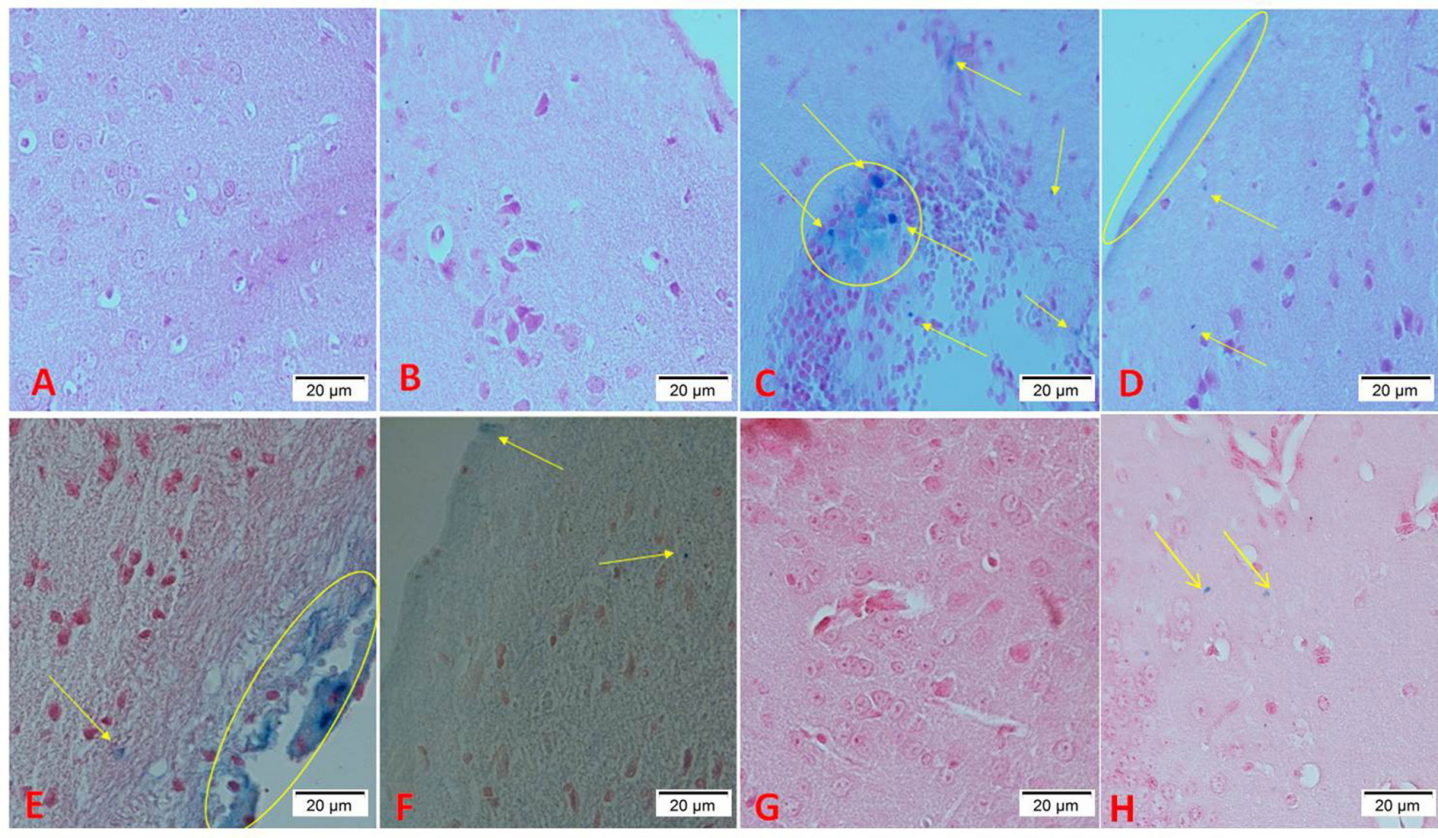
Perls staining of mice brain tissues [blue spots indicate iron deposition, indicated by arrows and circles; (magnification ×40)]. **(A)** Control group, normal saline-receiving group; **(B)** normal saline and DFO-receiving group; **(C)** iron dextran and normal saline-receiving group; **(D)** iron dextran and DFO-receiving group; **(E)** low dose caffeic acid-receiving group (30 mg/kg/day); **(F)** high dose caffeic acid-receiving group (50 mg/kg/day); **(G)** low dose ferulic acid-receiving group (30 mg/kg/day); and **(H)** high dose ferulic acid-receiving group (50 mg/kg/day).

Figure 4 shows the results of Perls iron staining of tissue samples. Blue spots indicate iron deposition. Figures 4A,B shows the brain of groups A and B, respectively. As it is observed, there is no iron deposition in these groups. Figure 4C displays the iron-overloaded group. In this group, a large iron deposition is observed. DFO administration has significantly reduced iron deposition in the brain tissue (Figure 4D). Caffeic acid treatment significantly reduced iron deposition (Figures 4E,F). In the group treated with a lower dose of ferulic acid, the iron deposition rate was lower than its high dose (Figures 4G,H).

**Figure 5:**
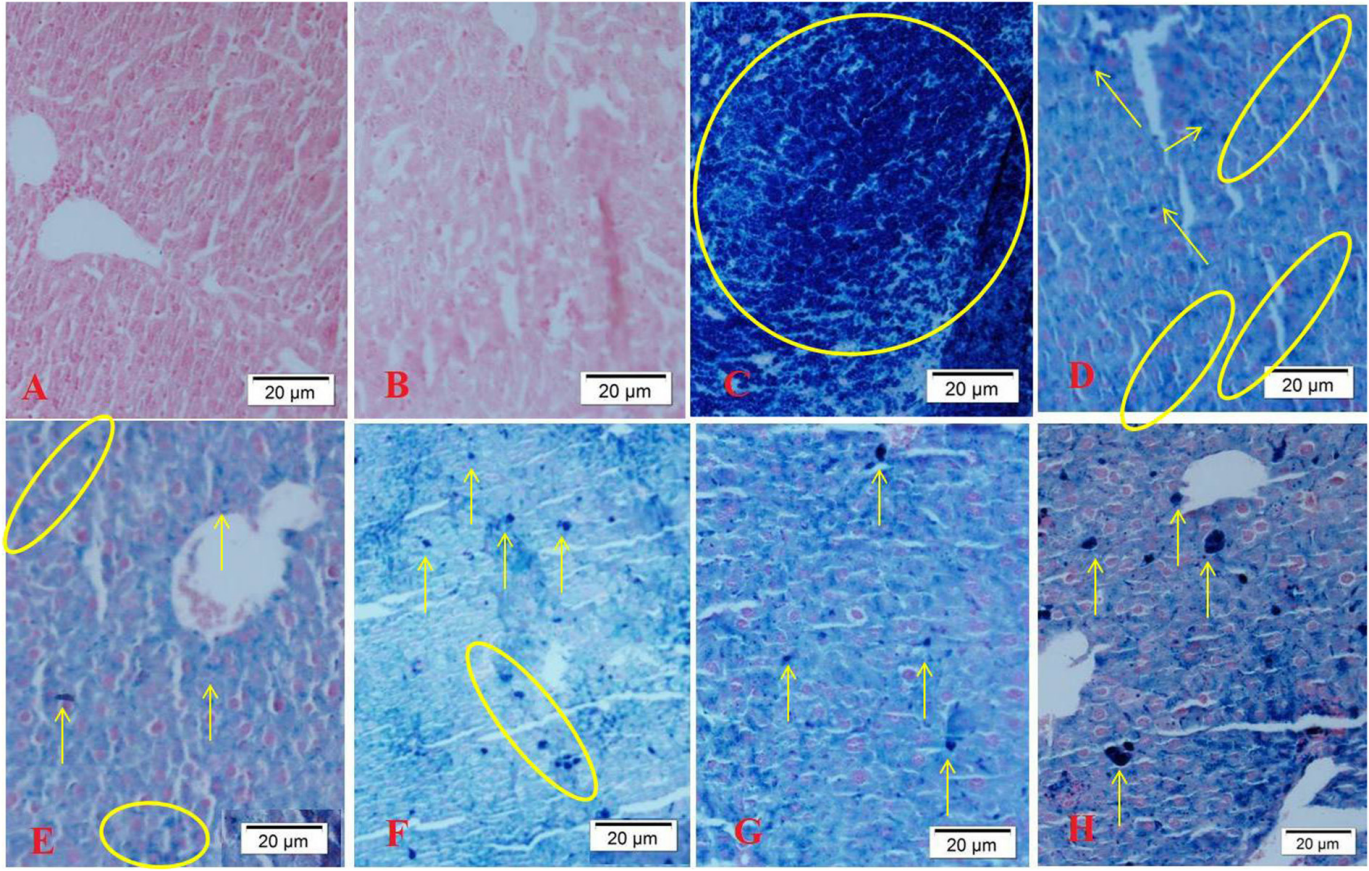
Perls staining of mice liver tissues (blue spots indicate iron deposition, indicated by arrows and circles; (magnification ×40). **(A)** Control group, normal saline-receiving group; **(B)** normal saline and DFO-receiving group; **(C)** iron dextran and normal saline-receiving group; **(D)** iron dextran and DFO-receiving group; **(E)** low dose caffeic acid-receiving group (30 mg/kg/day); **(F)** high dose caffeic acid-receiving group (50 mg/kg/day); **(G)** low dose ferulic acid-receiving group (30 mg/kg/day); and **(H)** high dose ferulic acid-receiving group (50 mg/kg/day).

In Figure 5, related to the liver tissue, it is clear that except for the two groups A and B, there is a high amount of iron sedimentation in iron-receiving groups. However, the administration of desferal or natural compounds ferulic acid and caffeic acid have greatly reduced the amount of iron in the liver tissue compared with group C. The highest reduction of liver iron is observed in groups E and G treated with ferulic acid and caffeic acid, respectively.

### The catalase enzyme activity

**Figure 6:**
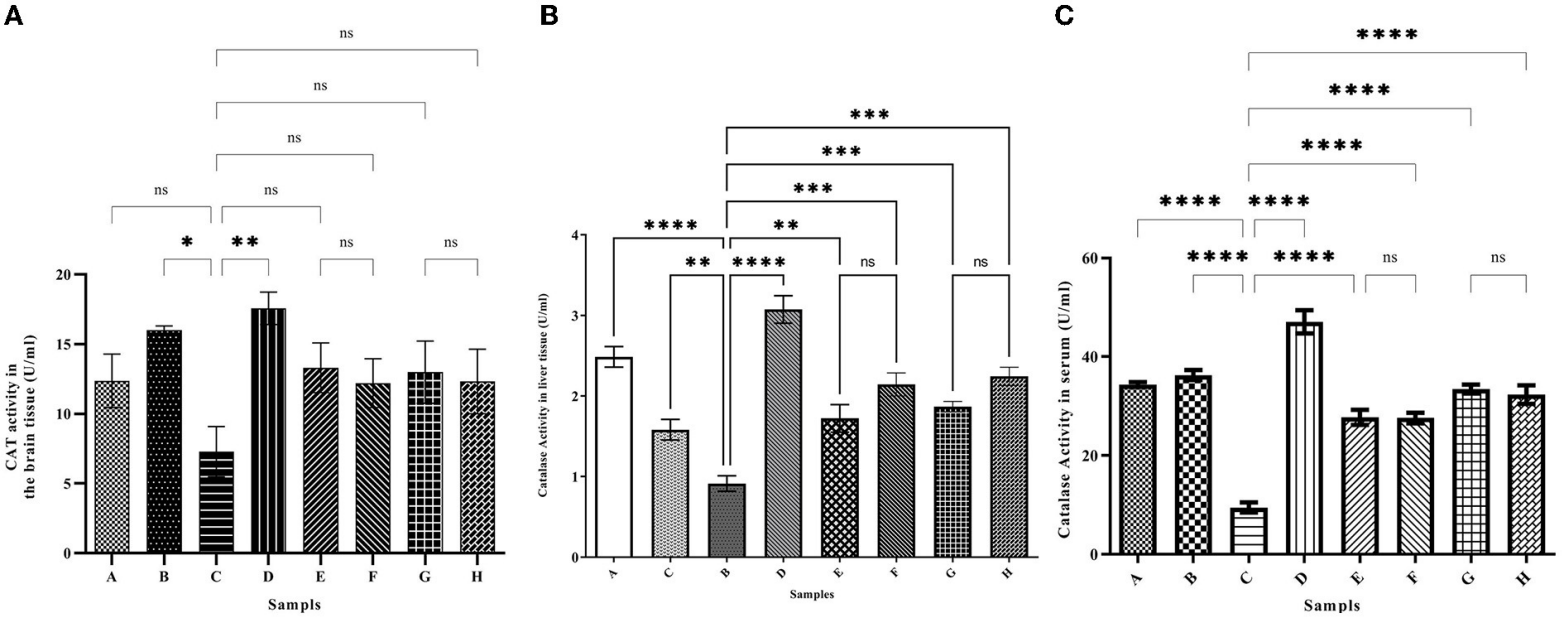
The activity level of the catalase (CAT) antioxidant enzyme in the brain **(A)**, liver **(B)**, and serum **(C)**. Iron greatly reduces the activity of catalase. Desferal or caffeic acid and ferulic acid improve the activity of catalase enzyme with their antioxidant effect. Group B: normal saline and DFO-receiving group; Group C: iron dextran and normal saline-receiving group; Group D: iron dextran and DFO-receiving group; Group E: low dose caffeic acid-receiving group (30 mg/kg/day); Group F: high dose caffeic acid-receiving group (50 mg/kg/day); Group G: low dose ferulic acid-receiving group (30 mg/kg/day); and Group H: high dose ferulic acid-receiving group (50 mg/kg/day) (Mean ± SD; ^*^p ≤ 0.05, ^**^p ≤ 0.01, ^***^p ≤ 0.001, and ^****^p ≤ 0.0001).

To investigate the oxidative status of the tissues, the activity level of the antioxidant enzyme catalase was investigated in the brain, liver tissues, and serum. As shown in Figure 6, Iron intake caused a strong decrease in the catalase activity in both tissues and serum compared with the control group (Figures 6A–C) (6.71 ± 2.59, 1.58 ± 0.12, and 9.49 ± 1.02 U/ml, respectively). The catalase activity in the brain of the iron group decreased to 6.71 ± 2.59 U/ml compared with the control group (13.87 ± 0.19 U/ml). In group D, which was iron overloaded and then treated with the desferal catalase activity was increased significantly up to 17.8 ± 0.45 U/ml. Both concentrations of ferulic acid and caffeic acid increased the catalase activity to the normal level in the control group. Changes in the activity of the antioxidant enzyme catalase in the liver were similar to those in the brain (Figure 6B). In the iron overloaded group (Group C), the catalase activity was decreased significantly in the liver compared to group C. However, the administration of desferal or ferulic acid and caffeic acid greatly improved catalase activity. The highest CAT activity was found in group D (the iron overloaded group that was treated by DFO) (3.07 ± 0.17 U/ml). According to Figure 6C, the minimum CAT activity in serum was obtained in group C (9.49 ± 1.02 U/ml), and the highest one was found in group D (47.08 ± 2.37 U/ml). In treated groups with natural products, CAT activity was increased significantly.

## Discussion

Medicinal plants contain various compounds, i.e., phenols, flavonoids, coumarin, and anthocyanins. Studies have shown that these compounds have antioxidant and chelating capacities (16, 32, 33). Plant extracts can be a new source of chelators and a good candidate for the treatment of iron overload-related diseases. Our previous studies have shown that plant extracts have the antioxidant and iron-chelating capacities *in vitro* and *in vivo* (26, 32).

Since mammals' bodies need iron, complex mechanisms have been developed to absorb, transport, and store iron in their bodies. Existing mechanisms safely inhibit iron overdose. Fe^2+^ can enter the Fenton reaction and react with hydrogen peroxide to produce a hydroxyl radical. The resulting reactive oxygen species (ROS) can damage biomolecules and lead to various diseases (34, 35). A quick treatment is the use of iron chelators (34, 36). Various studies have shown that iron can cross the blood-brain barrier (BBB) and accumulate in neurons (37). On the other hand, the total brain iron concentration increases with age and leads to diseases, such as Alzheimer's, Parkinson's, and Huntington's disease (34, 38). Studies have shown that some natural compounds have antioxidant and iron-chelating capacities *in vitro* (8, 26). The results of the present study have shown that ferulic acid and caffeic acid have iron-chelating capacity *in vivo*. Natural compounds ferulic acid and caffeic acid could reduce iron overload in the brain, liver, and serum. Studies show that ferulic acid can cross the blood-brain barrier (BBB) (28, 29). These compounds showed stronger chelating capacity than DFO (a common iron chelator). As the results of the present study showed, the total brain iron of the iron overload group increased, which confirms that iron crosses the blood-brain barrier. Our previous study have shown that naringin reduced the brain and serum iron levels in iron-overloaded mice (8). Mandel et al. (37) showed that green tea catechins have iron-chelating, antioxidant, and anti-inflammatory properties, and can penetrate the blood-brain barrier and protect against nerve death in a wide range of cellular and animal models of neurological diseases. Natural compounds such as coumarin and epigallocatechin gallate have iron-chelating capacity and reduce the negative effects of iron in the brain (39). In the DFO, caffeic acid, and ferulic acid-treated groups, the total iron was decreased in the brain and liver. The results of the present study also showed treatment with natural compounds reduced brain and liver iron deposition better than DFO. Reducing iron levels depends on the dose of natural compounds. Various studies have shown that natural compounds reduce oxidative stress in the brain tissue and reduce the production of ROS. Our results show that there are no significant differences between iron chelation of DFO, caffeic acid, and ferulic acid (Figures 1–3). Previous studies have shown that hesperidin and coumarin chelate iron are less than DFO (2). Other studies on naringin have shown that the total iron in the brain was decreased by consuming 60 mg/kg/day of naringin. DFO chelates iron better than 30 mg/kg/day of naringin (8). Due to the negative effects of DFO, the use of natural compounds as iron chelators is justified. Other studies have shown some natural products, such as CaA, FA, Berberine, and Vanillic acid, reduced iron content in the heart or liver significantly (2, 40–42). Resveratrol reduces oxidative stress in the brains of treated rats (43). As iron overload leads to oxidative stress, as shown in Figures 6A–C, the CAT activity was decreased significantly in group C (iron overloaded group) compared with group A. According to our results, DFO significantly increased CAT activity (Figures 6A–C). FA and CaA increased CAT activity in the brain, liver, and serum of the treatment mice (Figures 6A–C). Previous studies have shown that the antioxidant enzyme activity was affected by iron overload; and natural products and DFO increased the antioxidant enzyme activity (2, 40, 41).

A study investigated the neuroprotective and chelating capacities of green tea catechins *in vitro* and *in vivo*. The results showed that these natural compounds reduce the negative effects of iron and reduce the risk of developing Alzheimer's and Parkinson's diseases (37, 44). There are various reports that different natural compounds, such as curcumin, baicalein, apocynin, and quercetin, have iron-chelating and brain-protective properties (45). Collectively, iron is an important factor in the normal functioning of neurons, but iron accumulation in the brain causes many diseases. Iron therapy is a new strategy for treating diseases, such as Alzheimer's and Parkinson's diseases. It is possible to prevent the development of iron-related diseases in the brain by early detection of iron-overload conditions and the use of natural iron chelators (46).

## Conclusion

The results of the present study showed that caffeic acid and ferulic acid are strong iron-chelating agents compared with DFO in the brain and liver of iron-overloaded mice. In addition, the results of this study show that ferulic acid and caffeic acid have direct iron chelating and scavenging activities *in vitro*. It is recommended to investigate the effects of caffeic acid and ferulic acid on Alzheimer's and Parkinson's diseases in future clinical studies.

## Data availability statement

The original contributions presented in the study are included in the article/supplementary material, further inquiries can be directed to the corresponding author.

## Ethics statement

The animal study was reviewed and approved by Ethics Committee of Golestan University of Medical Sciences, Gorgan, Iran (Approval number: IR.GOUMS.REC.1397.179).

## Author contributions

MA performed the experiments. MK contributed to the conception and design of the study, verified the analytical methods, wrote the first draft of the manuscript, contributed to the interpretation of the results, and supervised the project. MJ performed the statistical analysis and designed the figures. All authors contributed to the manuscript revision and read, and approved the submitted version.

## Funding

This study was supported by the Golestan University of Medical Sciences (Gorgan, Iran) under Grant Number 110246.

## Conflict of interest

The authors declare that the research was conducted in the absence of any commercial or financial relationships that could be construed as a potential conflict of interest.

## Publisher's note

All claims expressed in this article are solely those of the authors and do not necessarily represent those of their affiliated organizations, or those of the publisher, the editors and the reviewers. Any product that may be evaluated in this article, or claim that may be made by its manufacturer, is not guaranteed or endorsed by the publisher.

## References

[1] Sobotka T Whittaker P Sobotka J Brodie R Wander D Robl M. Neurobehavioral dysfunctions associated with dietary iron overload. Physiol Behav. (1996) 59:213–9. 10.1016/0031-9384(95)02030-6 8838597

[2] Aalikhani M Safdari Y Jahanshahi M Alikhani M Khalili M. Comparison between hesperidin, coumarin, and deferoxamine iron chelation and antioxidant activity against excessive iron in the iron overloaded mice. Front Neurosci. (2022) 15:811080. 10.3389/fnins.2021.811080 35177961 PMC8846322

[3] Yun S He X Zhang W Chu D Feng C. Alleviation effect of grape seed proanthocyanidins on neuronal apoptosis in rats with iron overload. Biol Trace Elem Res. (2020) 194:210–20. 10.1007/s12011-019-01766-8 31236816

[4] Cobley JN Fiorello ML Bailey DM. 13 reasons why the brain is susceptible to oxidative stress. Redox Biol. (2018) 15:490–503. 10.1016/j.redox.2018.01.008 29413961 PMC5881419

[5] Piñero DJ Jones BC Beard JL. Variations in dietary iron alter behavior in developing rats. J Nutr. (2001) 131:311–8. 10.1093/jn/131.2.311 11160552

[6] Kim J-J Kim Y-S Kumar V. Heavy metal toxicity: an update of chelating therapeutic strategies. J Trace Elem Med Biol. (2019) 54:226–31. 10.1016/j.jtemb.2019.05.003 31109617

[7] Bami E Ozakpinar OB Ozdemir-Kumral ZN Köroglu K Ercan F Cirakli Z. Protective effect of ferulic acid on cisplatin induced nephrotoxicity in rats. Environ Toxicol Pharmacol. (2017) 54:105–11. 10.1016/j.etap.2017.06.026 28704751

[8] Jahanshahi M Khalili M Margedari A. Naringin chelates excessive iron and prevents the formation of amyloid-beta plaques in the hippocampus of iron-overloaded mice. Front Pharmacol. (2021) 12:651156. 10.3389/fphar.2021.651156 34276359 PMC8283124

[9] De Lima M Polydoro NM Laranja M Bonatto DC Bromberg F Moreira E. Recognition memory impairment and brain oxidative stress induced by postnatal iron administration. Eur J Neurosci. (2005) 21:2521–8. 10.1111/j.1460-9568.2005.04083.x 15932609

[10] Kaur D Yantiri F Rajagopalan S Kumar J Mo JQ Boonplueang R. Genetic or pharmacological iron chelation prevents MPTP-induced neurotoxicity in vivo: a novel therapy for Parkinson's disease. Neuron. (2003) 37:899–909. 10.1016/S0896-6273(03)00126-0 12670420

[11] Bar-Am O Amit T Kupershmidt L Aluf Y Mechlovich D Kabha H. Neuroprotective and neurorestorative activities of a novel iron chelator-brain selective monoamine oxidase-A/monoamine oxidase-B inhibitor in animal models of Parkinson's disease and aging. Neurobiol Aging. (2015) 36:1529–42. 10.1016/j.neurobiolaging.2014.10.026 25499799

[12] Sripetchwandee J Wongjaikam S Krintratun W Chattipakorn N Chattipakorn SC. P4-021: Combined iron chelator and antioxidant therapy effectively diminishes the dendritic loss, Alzheimer's pathology and brain mitochondrial dynamic disruption in rats with chronic iron overload. Alzheimers Dement. (2016) 12:P1022–3. 10.1016/j.jalz.2016.06.2110 27403880

[13] Heli H Mirtorabi S Karimian K. Advances in iron chelation: an update. Expert Opin Ther Pat. (2011) 21:819–56. 10.1517/13543776.2011.569493 21449664

[14] Piga A Roggero S Salussolia I Massano D Serra M Longo F. Deferiprone. Ann N Y Acad Sci. (2010) 1202:75–8. 10.1111/j.1749-6632.2010.05586.x 20712776

[15] Khalili M Ebrahimzadeh MA Kosaryan M. In vivo iron-chelating activity and phenolic profiles of the angel's wings mushroom, pleurotus porrigens (Higher Basidiomycetes). Int J Med Mushrooms. (2015) 17:847–56. 10.1615/IntJMedMushrooms.v17.i9.50 26756297

[16] Khalili M Ebrahimzadeh MA Kosaryan M Abbasi A Azadbakht M. Iron chelation and liver disease healing activity of edible mushroom (Cantharellus cibarius), in vitro and in vivo assays. RSC Adv. (2015) 5:4804–10. 10.1039/C4RA11561A

[17] Zhao Z Moghadasian MH. Chemistry, natural sources, dietary intake and pharmacokinetic properties of ferulic acid: a review. Food Chem. (2008) 109:691–702. 10.1016/j.foodchem.2008.02.039 26049981

[18] Kumar N Pruthi V. Potential applications of ferulic acid from natural sources. Biotechnology Reports. (2014) 4:86–93. 10.1016/j.btre.2014.09.002 28626667 PMC5466124

[19] Balasubashini MS Rukkumani R Viswanathan P Menon VP. Ferulic acid alleviates lipid peroxidation in diabetic rats. Phytother Res. (2004) 18:310–4. 10.1002/ptr.1440 15162367

[20] Yeh Y-H Y-Lee T H-Hsieh S Hwang D-F. Dietary caffeic acid, ferulic acid and coumaric acid supplements on cholesterol metabolism and antioxidant activity in rats. J Food Drug Anal. (2009) 17. 10.38212/2224-6614.2292

[21] Khan FA Maalik A Murtaza G. Inhibitory mechanism against oxidative stress of caffeic acid. J Food Drug Anal. (2016) 24:695–702. 10.1016/j.jfda.2016.05.003 28911606 PMC9337298

[22] Murtaza G Sajjad A Mehmood Z Shah SH Siddiqi AR. Possible molecular targets for therapeutic applications of caffeic acid phenethyl ester in inflammation and cancer. J Food Drug Anal. (2015) 23:11–8. 10.1016/j.jfda.2014.06.001 28911433 PMC9351751

[23] Sato Y Itagaki S Kurokawa T Ogura J Kobayashi M Hirano T. In vitro and in vivo antioxidant properties of chlorogenic acid and caffeic acid. Int J Pharm. (2011) 403:136–8. 10.1016/j.ijpharm.2010.09.035 20933071

[24] Jayanthi R Subash P. Antioxidant effect of caffeic acid on oxytetracycline induced lipid peroxidation in albino rats. Indian J Clin Biochem. (2010) 25:371–5. 10.1007/s12291-010-0052-8 21966107 PMC2994573

[25] Agunloye OM Oboh G Ademiluyi AO Ademosun AO Akindahunsi AA Oyagbemi AA. Cardio-protective and antioxidant properties of caffeic acid and chlorogenic acid: mechanistic role of angiotensin converting enzyme, cholinesterase and arginase activities in cyclosporine induced hypertensive rats. Biomed Pharmacother. (2019) 109:450–8. 10.1016/j.biopha.2018.10.044 30399581

[26] Ebrahimzadeh MA Safdari Y Khalili M. Antioxidant activity of different fractions of methanolic extract of the golden chanterelle mushroom Cantharellus cibarius (higher basidiomycetes) from Iran. Int J Med Mushrooms. (2015) 17:557–65. 10.1615/IntJMedMushrooms.v17.i6.60 26349513

[27] Charan J Kantharia N. How to calculate sample size in animal studies? J Pharmacol Pharmacother. (2013) 4:303. 10.4103/0976-500X.119726 24250214 PMC3826013

[28] Wang E-J Wu MY Lu JH. Ferulic acid in animal models of Alzheimer's disease: a systematic review of preclinical studies. Cells. (2021) 10:2653. 10.3390/cells10102653 34685633 PMC8534433

[29] Koh PO. Ferulic acid attenuates the injury-induced decrease of protein phosphatase 2A subunit B in ischemic brain injury. PLoS ONE. (2013) 8:e54217. 10.1371/journal.pone.0054217 23349830 PMC3547913

[30] Khalili M Ebrahimzadeh MA. A review on antioxidants and some of their common evaluation methods. J Maz Univ Med Sci. (2015) 24:188–208.

[31] Rebouche CJ Wilcox CL Widness JA. Microanalysis of non-heme iron in animal tissues. J Biochem Biophys Methods. (2004) 58:239–51. 10.1016/j.jbbm.2003.11.003 15026210

[32] Ebrahimzadeh MA Khalili M Dehpour AA. Antioxidant activity of ethyl acetate and methanolic extracts of two marine algae, nannochloropsis oculata and Gracilaria gracilis -an in vitro assay. Braz J Pharm Sci. (2018) 54:e17280. 10.1590/s2175-97902018000117280

[33] Loizzo MR Tundis R Bonesi M Menichini F Mastellone V Avallone L. Radical scavenging, antioxidant and metal chelating activities of Annona cherimola Mill. (cherimoya) peel and pulp in relation to their total phenolic and total flavonoid contents. J Food Compost Anal. (2012) 25:179–84. 10.1016/j.jfca.2011.09.002

[34] Fasae KD Abolaji AO Faloye TR Odunsi AY Oyetayo BO Enya JI. Metallobiology and therapeutic chelation of biometals (copper, zinc and iron) in Alzheimer's disease: limitations, and current and future perspectives. J Trace Elem Med Biol. (2021) 67:126779. 10.1016/j.jtemb.2021.126779 34034029

[35] Truong DH Nhung NTA Dao DQ. Iron ions chelation-based antioxidant potential vs. pro-oxidant risk of ferulic acid: A DFT study in aqueous phase. Comput Theor Chemist. (2020) 1185:112905. 10.1016/j.comptc.2020.112905

[36] Badria FA Ibrahim AS Badria AF Elmarakby AA. Curcumin attenuates iron accumulation and oxidative stress in the liver and spleen of chronic iron-overloaded rats. PLoS ONE. (2015) 10:e0134156. 10.1371/journal.pone.0134156 26230491 PMC4521784

[37] Mandel S Amit T Reznichenko L Weinreb O Youdim MB. Green tea catechins as brain-permeable, natural iron chelators-antioxidants for the treatment of neurodegenerative disorders. Mol Nutr Food Res. (2006) 50:229–34. 10.1002/mnfr.200500156 16470637

[38] Bruehlmeier M Leenders KL Vontobel P Calonder C Antonini A Weindl A. Increased cerebral iron uptake in Wilson's disease: a 52Fe-citrate PET study. J Nucl Med. (2000) 41:781–7. 10.2967/jnumed.118.212803 10809192

[39] Mandel S Amit T Bar-Am O Youdim MB. Iron dysregulation in Alzheimer's disease: multimodal brain permeable iron chelating drugs, possessing neuroprotective-neurorescue and amyloid precursor protein-processing regulatory activities as therapeutic agents. Prog Neurobiol. (2007) 82:348–60. 10.1016/j.pneurobio.2007.06.001 17659826

[40] Alikhani M Aalikhani M Khalili M. Reduction of iron toxicity in the heart of iron-overloaded mice with natural compounds. Eur J Pharmacol. (2022) 924:174981. 10.1016/j.ejphar.2022.174981 35487255

[41] Aalikhani M Alikhani M Jahanshahi M Elyasi L Khalili M. Berberine is a promising alkaloid to attenuate iron toxicity efficiently in iron-overloaded mice. Nat Prod Commun. (2022) 17:29522. 10.1177/1934578X211029522

[42] Jahanshahi M Khalili M Margdari A Aalikhani M. Naringin is a promising natural compound for therapy of iron-overload disorders. Braz J Pharm Sci. (2022) 58:1–7. 10.1590/s2175-97902022e19409

[43] Mokni M Elkahoui S Limam F Amri M Aouani E. Effect of resveratrol on antioxidant enzyme activities in the brain of healthy rat. Neurochem Res. (2007) 32:981–7. 10.1007/s11064-006-9255-z 17401679

[44] Mandel SA Amit T Weinreb O Reznichenko L Youdim MB. Simultaneous manipulation of multiple brain targets by green tea catechins: a potential neuroprotective strategy for Alzheimer and Parkinson diseases. CNS Neurosci Ther. (2008) 14:352–65. 10.1111/j.1755-5949.2008.00060.x 19040558 PMC6493995

[45] Davinelli S Di Marco R Bracale R Quattrone A Zella D Scapagnini G. Synergistic effect of L-Carnosine and EGCG in the prevention of physiological brain aging. Curr Pharm Des. (2013) 19:2722–7. 10.2174/1381612811319150007 23092324

[46] Zecca L Youdim MBH Riederer P Connor JR Crichton RR. Iron, brain ageing and neurodegenerative disorders. Nat Rev Neurosci. (2004) 5:863–73. 10.1038/nrn1537 15496864

